# Infection and atherosclerosis: TLR-dependent pathways

**DOI:** 10.1007/s00018-020-03453-7

**Published:** 2020-01-30

**Authors:** Bowei Li, Yuanpeng Xia, Bo Hu

**Affiliations:** Department of Neurology, Union Hospital, Tongji Medical College, Huazhong University of Science and Technology, Wuhan, 430022 China

**Keywords:** Infection, Atherosclerosis, Toll-like receptors, *Chlamydia pneumoniae*, *Porphyromonas gingivalis*

## Abstract

Atherosclerotic vascular disease (ASVD) is a chronic process, with a progressive course over many years, but it can cause acute clinical events, including acute coronary syndromes (ACS), myocardial infarction (MI) and stroke. In addition to a series of typical risk factors for atherosclerosis, like hyperlipidemia, hypertension, smoking and obesity, emerging evidence suggests that atherosclerosis is a chronic inflammatory disease, suggesting that chronic infection plays an important role in the development of atherosclerosis. Toll-like receptors (TLRs) are the most characteristic members of pattern recognition receptors (PRRs), which play an important role in innate immune mechanism. TLRs play different roles in different stages of infection of atherosclerosis-related pathogens such as *Chlamydia pneumoniae *(*C. pneumoniae*)*,* periodontal pathogens including *Porphyromonas gingivalis *(*P. gingivalis*)*, Helicobacter pylori *(*H. pylori*) and *human immunodeficiency virus* (HIV). Overall, activation of TLR2 and 4 seems to have a profound impact on infection-related atherosclerosis. This article reviews the role of TLRs in the process of atherosclerosis after *C. pneumoniae* and other infections and the current status of treatment, with a view to providing a new direction and potential therapeutic targets for the study of ASVD.

## Introduction

Atherosclerotic vascular disease (ASVD) is a chronic process, with a progressive course over many years, but it can cause acute clinical events, including acute coronary syndromes (ACS), myocardial infarction (MI) and stroke [1]. Hypertension, hypercholesterolemia and other continuous effects or vascular bifurcation disorder blood flow shear force lead to endothelial cells (ECs) dysfunction or anatomical damage. Lipids (mainly cholesterol and cholesterol esters) in the blood enter the arterial wall in the form of lipoproteins from the damaged ECs, causing local inflammation of the vessels [2]. Pathological high expression of P-selectin and vascular cell adhesion molecule-1 (VCAM-1) in injured ECs mediates the adhesion of leukocytes (mainly lymphocytes and monocytes) in the blood and infiltrates into the endothelium [3]. Inflammatory factors can stimulate monocytes to chemotaxis to the vascular wall and differentiate into macrophages, and form foam cells through phagocytosis of modified lipoproteins [such as oxidized low-density lipoprotein (ox-LDL)] [4]. With the development of inflammation, activated leukocytes and vascular ECs can release fibroblast growth regulating factor (FGF), induce the phenotype change of vascular smooth muscle cells (SMCs), migrate from the middle membrane through the inner elastic layer to the subintimal of arteries, proliferate and express a large number of cytokines and adhesion factors [5]. In the late stage of atherosclerosis, inflammatory cytokines and matrix metalloproteinases (MMP) can degrade extracellular matrix proteins, which make the plaque easy to rupture. In addition, inflammatory cells secrete vascular growth factors (VGF), which can promote the formation of blood vessels in the plaque, and eventually lead to MI or stroke [6].

Although the traditional risk factors of atherosclerosis, such as hyperlipidemia, hypertension, smoking, have been efficaciously cut down, the occurrence of atherosclerotic disease is still high. In addition, 30–50% of patients lack these typical risk factors, suggesting that other unknown factors are related to the pathogenesis [7]. Atherosclerosis was once thought to be a simple lipid accumulation lesion, but over the past decades emerging evidence has reported that atherosclerosis is a chronic inflammatory disease, suggesting that chronic infection plays a critical role in the development of atherosclerosis [8]. Fabricant and colleagues induced chicken atherosclerosis by infection with Marek’s disease virus, and vaccination can prevent atherosclerotic changes caused by Marek’s disease virus, which has aroused great interest in the study of the infectivity of atherosclerosis [9]. Subsequently a series of infectious agents were proved to be closely related to atherosclerosis, including *Chlamydia pneumoniae *(*C. pneumoniae*) [10], *Porphyromonas gingivalis *(*P. gingivalis*) [11], *Helicobacter pylori *(*H. pylori*) [12], *cytomegalovirus* (CMV) [13], *Epstein-Barr virus* (EBV) [14], *human immunodeficiency virus* (HIV) [15], *herpes simplex virus-1* (HSV-1) [16], HSV-2 [17] and *hepatitis C virus* (HCV) [18]. A large number of studies have detected the presence of bacterial or viral pathogens in atherosclerotic plaques, suggesting that these pathogens can invade, lurk or replicate in cells, so as to play a direct role in the local plaque [19, 20]. The mechanisms by which these pathogens promote atherosclerosis are by promoting ECs inflammatory responses and macrophage-derived foam cell formation, as well as increasing SMCs proliferation and inhibiting their apoptosis. However, in most animal experiments, microbial infection can accelerate the progression of atherosclerotic plaque on the basis of dietary risk factors or genetic susceptibility or vascular damage, but it cannot be used as a single pathogenic factor to cause plaque formation. In addition, in epidemiological studies, a variety of infectious microbes is associated with atherosclerotic vascular disease, but several clinical trials have shown that anti-infective therapy is ineffective in reducing atherosclerotic cardiovascular events. Therefore, the mechanism involved in infection to accelerate atherosclerosis has not been definitively identified until the exciting discovery related to signaling via toll-like receptors (TLRs) once again triggers a strong interest in immune defense mechanisms.

TLRs are the most characteristic members of pattern recognition receptors (PRRs) and play vital roles in innate immunity mechanisms [21]. Located on the cell membrane or in the cytoplasm, PRRs can recognize conserved microbial structures called pathogen-associated molecular patterns (PAMPs) such as lipopolysaccharides (LPS) released from Gram-negative bacteria or viral RNA, as well as host biomolecules associated with cell injury or necrosis called danger-associated molecular patterns (DAMPs) such as high mobility group protein B1 (HMGB1) [22]. TLRs orchestrate both pathogen-specific and cell type-specific host immune responses to fight infections. They play such a central role in initiating immune responses to a variety of pathogens that it is not surprising that in some cases inflammatory diseases such as atherosclerosis are caused by inappropriate activation of them [23]. TLRs are considered to be an important factor in the pathogenesis of atherosclerosis. TLR2-TLR1 heterodimer and TLR2-TLR6 heterodimer contributed to atherosclerosis in ApoE gene-knockout (ApoE^−/−^) mice and LDL receptor deficient (LDLR^−/−^) mice [24, 25]. TLR4 deficiency improved the atherosclerosis index in ApoE^−/−^ and LDLR^−/−^ mice [26, 27]. In addition, in endoplasmic TLRs, TLR3 can regulate the activities of MMP-2 and MMP-9 in macrophages, thus affecting the instability of atherosclerotic plaque [28]. In response to vascular injury, foam cell accumulation and lesion formation, and in LDLR^−/−^ mice, TLR9 has been observed to spread inflammation [29]. Interestingly, there is evidence that some endoplasmic TLRs can improve the occurrence of atherosclerosis. It has been observed that TLR3 has a protective effect on the vascular wall after mechanical and hypercholesterolemia-induced arterial injury [30]. TLR7 activation inhibited the activation of inflammatory macrophages and the production of cytokines [31]. In addition, TLR9 gene deletion aggravated the atherosclerosis of ApoE^−/−^ mice fed with high-fat-diet (HFD) and a TLR9 agonist reduced the severity of the disease [32]. The mechanisms of atherogenesis induced by TLRs include the dysfunction of vascular cells, the recruitment of macrophages and other immune cells to the site of vascular injury, the formation of foam cells, and the instability of plaques, while the anti-atherosclerotic effect of TLRs is more in line with its evolutionary conservative function. To expand the positive effects of TLRs in identifying pathogens and minimize the negative ones should be the goal of researchers in the field of TLRs and atherosclerosis.

The present review aims to summarize the latest progression and focus on the role of TLRs between microbial infection and atherosclerosis, hoping to provide a reference and treatment for strategy of atherosclerosis. We pay special attention to three research areas: (1) the biological characteristics and functions of TLRs are systematically summarized; (2) advances in research on the relationship between different pathogens and atherosclerosis, and the role or potential role of TLRs in inducing or accelerating atherosclerosis; (3) current status and potential therapeutic targets of antibiotic and vaccine therapy for infection and targeting TLRs in atherosclerotic diseases. Finally, we summarize the full text, trying to explore the future research direction and potential drug treatment targets of atherosclerosis.

## Biological characteristics of TLRs

### Classification and structure of TLRs

The research history of TLRs dates back to the last century and as early as 1991 the homology between the Drosophila toll receptors and the interleukin-1 (IL-1) receptors was identified by Gay and Keith [33]. In 1994, Nomura et al. discovered the first sequence of a mammalian TLR homologue [34]. In 1996, it was discovered to be involved in anti-fungal immunity by Lemaitre et al. [35]. In 1997, Medzhitov et al. identified TLRs as vertebrate homologs of the Drosophila spp. determining Drosophila dorsal ventral polarity during Drosophila embryo development [36]. They also demonstrated that chimera of the intracellular domain of TLRs and the extracellular domain of CD4 activate nuclear factor kappa-B (NF-κB), which thereby raises the expression of inflammatory cytokines IL-1, IL-6, IL-8 and costimulatory molecules B7.1. and eventually activates T lymphocytes. In 1998, it was recognized that there is a complete set of mammalian TLRs that respond to microbial ligands, while the long-term search for LPS bacterial receptors is TLR4 [37]. As of this writing, there are 13 TLRs identified in mammalian species of which TLR11 is non-functional and TLR12-13 are only expressed in mice [38]. Although TLRs are widely expressed in mammalian cells such as dendritic cells (DCs), macrophages, neutrophils, monocytes, lymphocytes, fibroblasts, epithelial cells, ECs, nerve cells and so on, each cell type includes a specific group of TLRs, which play a unique role in identifying PAMPs or DAMPs and mediate the immune response [39]. Mammalian TLRs can be divided into cell membrane and intracellular receptors according to its location. One group consisted of TLR1, 2, 5, 6, 10 and 11, which were expressed on the cell surface and mainly recognized microbial membrane components such as LPS; the other group consisted of TLR3, 7, 8, 9, 12 and 13, which were only expressed in intracellular vesicles and recognized microbial nucleic acids, and TLR4 localizes to the cell surface and intracellular vesicles [40]. According to sequence analysis and three-dimensional structure, TLR can be further divided into three domains TLR (TLR1, 2, 4, 6, 10) interacting with lipid molecules such as LPS and lipoproteins, and single domain TLR (TLR3, 5, 7, 8, 9) interacting with hydrophilic ligands such as nucleic acids [41]. These TLRs contain three main domains: (1) extracellular domain of hydrophobic leucine rich regions, 16–28 leucine rich repeats (LRRs), which mainly perform the function of recognizing receptors and binding with other co receptors to form receptor complexes. (2) transmembrane region; and (3) cytoplasmic region highly homologous with the cytoplasmic region of the members of the IL-1R family (IL-1R-mediated signal transduction system and mechanism are similar to that of Drosophila), which is called the toll-IL-1 receptor domain (TIR domain) [42]. TIR has homophilic interaction, by which downstream signal molecules containing TIR, such as MyD88 (myelid differentiation factor 88) and TIRAP (TIR domain containing adapter protein), are recruited to form signal complexes, which play an important role in TLR signaling transmission. See below for details.

### Endogenous and exogenous ligands of TLRs

TLR ligands are extremely extensive, each type of them has a series of ligands with a certain degree of specificity. Most TLRs are homodimers, but some are heterodimers. TLR2 has been proved to be able to sense lipopeptides: it is heterodimerized with TLR1 to recognize triacyl lipopeptides from Gram-negative bacteria and mycoplasma, or heterodimerized with TLR6 to recognize diacyl lipopeptides from Gram-positive bacteria and mycoplasma. The structure of the two dimers was finally solved in 2007 [43, 44]. TLR2 has also been proved to recognize many other non-lipopeptide PAMPs from various etiologies. TLR4 recognizes LPS, lipoteichoic acid (LTA), envelope glycoprotein and protein F on the plasma membrane, ox-LDL, Tenascin C, fibronectin extra domain A (EDA) on the endosomes [45, 46]. Before TLR3 was discovered in 2001, it was thought that TLRs were not involved in antiviral reactions. In 2001, TLR3 was proved to be able to recognize double stranded RNA (dsRNA), which is the main component of many viruses, and mediates the activation of NF-κB and type I IFN signaling pathway. TLR3 was then identified to sense small interfering RNA (siRNA) and self RNA from damaged cells [47]. In the same year, TLR5 was proved to be able to sense bacterial flagellin (a protein component of flagellin) [48]. Further studies showed that TLR5 can regulate the innate and adaptive response of bacteria in the intestinal tract [49]. In 2002, TLR7 was considered as another antiviral TLR because it can sense the synthetic chemical imiquimod, which is believed to stimulate antiviral response. Subsequently, in 2004, TLR7 and tlr8 were proved to be able to sense single stranded RNA (ssRNA). TLR9 has a specific response to CpG-rich hypomethylated DNA motifs. These motifs mainly exist in bacteria, mitochondria and fetal DNA, but rarely in adult DNA of vertebrates [50]. Recent studies have shown that TLR9 also reacts to herpesvirus DNA and the byproduct of Plasmodium falciparum, hemozoline [51]. Human TLR10 and TLR2 recognize the ligands of Listeria, while mouse TLR10 contains a termination codon, so it is a pseudogene. TLR10 also responded to influenza A virus infection. TLR11 in mice has been proved to be able to detect a component of urinary pathogenic bacteria, and cooperate with TLR12 in mice to bind with profilin protein of Toxoplasma gondii [52]. Mouse TLR13 has recently been shown to recognize bacterial ribosomal RNA [53]. All these findings confirm that TLRs are a large family of receptors, which can activate innate immunity and inflammation to deal with dangerous signals such as infection or tissue damage. The details showed in Table 1.

**Table 1:** Endogenous and exogenous ligands of TLRs

TLRs	Human	Mouse	Localization	Vascular target cells	Exogenous ligands	Origin of ligands	Endogenous ligands
TLR1	+	+	Extracellular	Monocytes, DCs, T, B and NK cells	Soluble factors (with TLR2)	*Neisseria meningitidis*	Necrotic cells
					Triacyl lipopeptides	Bacteria, mycobacteria	*ApoliproteinC III*
TLR2	+	+	Extracellular	Immune cells except T, B and NK cells	Atypical LPS	*Leptospira interrogans* and *P. gingivalis*	*Ox LDL*
					Envelope glycoproteins	Virus	Serum amyloid A
					Glycoinositolphospholipids	*Trypanozoma cruzi*	Amyloid beta
					Glycolipids	Treponema maltophilum	Versican
					Lipoarabinomannan	Mycobacteria	
					Lipoprotein (with TLR1)	Mycobacteria, *Borrelia burgdorferi*	
					Lipoprotein (with TLR6)	Bacteria	
					Lipoteichoic acid	Gram-positive bacteria	
					*Pam3CSK4*	Synthetic TLR2/TLR1 agonist	
					*PGN*	Gram-positive bacteria	
					Phenol-soluble modulin	*Staphylococcus aureus*	
					Phospholipomannan	*Candida albicans*	
					Porins	*Neisseria meningitidis*	
					Triacyl lipopeptides	Bacteria, mycobacteria	
					Yeast carbohydrates		
					Zymosan	Fungi	
TLR2/TLR4	+	+	Extracellular	Immune cells except T, B and NK cells	HSP60	*C. pneumoniae*	HSP60
						*P. gingivalis*	HSP70
							Gp96
							HMGB1
							*Hyaluronan fragment*
							*Biglycan*
TLR3	+	+	Intracellular	Immature DCs	*dsDNA*	Virus	*mRNA*
TLR3/TLR9	+	+	Intracellular	DCs		CMV	
TLR4	+	+	Extracellular	Immune cells except T, B and NK cells	*LPS*	Gram-negative bacteria	Lung surfactant protein-A
					Envelope glycoproteins	Virus	Tenascin C
					Envelope proteins	MMTV	*Fibrinogen*
					Fusion protein	RSV	*Fibronectin EDA*
					HSP60	*C. pneumoniae*	*Heparan sulphate*
					Lipoteichoic acids	Gram-positive bacteria	*Beta-defensin 2*
					Mannuronic acid polymer	Aeruginosa	*Minimally-modified LDL*
					Protein F	RSV Pseudomonas	*Ox LDL*
					Taxol	Plant	Amyloid beta peptide
TLR5	+	+	Extracellular	Myelogenous cells (monocyte macrophages)	Flagellin	Bacteria	
TLR6	+	+	Extracellular	Immune cells	Diacyl lipopeptides	Mycoplasma	Ox LDL
					Group B strep heat-labile soluble factor		
					Phenol-soluble modulin	*Staphylococcus aureus*	
TLR7	+	+	Intracellular	DCs	Loxoribine and bropirimine		
					Various synthetic compounds including imidazoquinoline		
TLR7/TLR8	+	+	Intracellular	DCs	ssRNA		
TLR7/TLR9	+	+	Intracellular	DCs			Nucleic acid-containing immune complexes
TLR9	+	+	Intracellular	DCs	HSV-2		
					Hypomethylated CpG motifs in microbial DNA		
TLR10	+	–	Unknown		Unknown		Unknown
TLR11	–	+	Extracellular	Nerve cells and immune cells	Uropathogenic bacteria		
TLR11/TLR12	–	+	Intracellular	Nerve cells and immune cells	Profilin		
TLR13	–	+	Intracellular	Nerve cells and immune cells	23S rRNA		

Expression of TLRs in humans and mice and expression in and out of cells. The endogenous and exogenous ligands of TLRs, as well as the source of exogenous ligands, are listed in detail. Italics indicate that a ligand associated with atherosclerosis has been identified

*DCs* dendritic cells, *LPS* lipopolysaccharide, *oxLDL* oxidized low-density lipoprotein, *LAM* lipoarabinomannan, *LTA* lipoteichoic acid, *PGN* peptidoglycan, *C. pneumoniaeChlamydia pneumonia,HSP* heat-shock protein, *HMGB1* high-mobility group box 1 protein, *dsRNA* double-stranded RNA, *MMTV* mouse mammary tumour virus, *RSV* respiratory syncytial virus, *ssRNA* single-stranded RNA

### TLR signaling pathways

The specific response initiated by an individual TLR depends on the recruitment of a single or specific binding adapter protein containing the TIR domain. These adaptors are MyD88, MyD88-adaptor-like (MAL, also known as TIRAP), TIR-domain-containing adaptor protein inducing IFNβ (TRIF; also known as TICAM1), TRIF-related adaptor molecule (TRAM; also known as TICAM2) and sterile α- and armadillo-motif-containing protein (SARM). SARM has now been shown to interact with TRIF and thereby interfere with TRIF function [54]. MyD88 is used by all TLRs (except TLR3) and IL-1 receptor family members, and transmits signals in NF-κB and MAP kinase activation and inflammatory cytokine induction. Among them, TLR1, 2, 4 and 6 recruit additional adapter MAL as a bridge between TIR domain and MyD88. TLR3 and 4 can also induce the production of type I IFN and inflammatory cytokines by using the MyD88-dependent pathway of TRIF. Compared with TLR3, TLR4 does not interact with TRIF directly, but needs TRAM to transmit its signal. In addition, recent studies have shown that TLR5 in intestinal epithelial cells recruits TRIF in addition to MyD88, which results in activation of NF-κB rather than IRF3. Therefore, TLR4 is the only one that recruits four adaptor proteins. It recruits TIRAP on the cell surface, promotes the aggregation of MyD88, and initiates a pathway dependent on MyD88. Then, TLR4 depends on the endocytosis of dynein and is transported to endoplasmic body, resulting in the formation of signal complex composed of endoplasmic body, TRAM and TRIF, thus initiating the TRIF-dependent pathway [55].

**Fig. 1:**
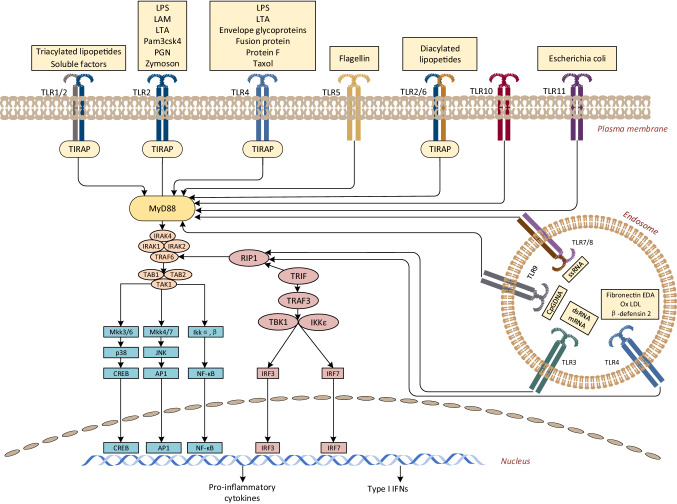
Mammalian TLR signaling pathway. TLR2 usually forms heterodimers with TLR1 or TLR6. On the cell membrane, TLR2-TLR1 and TLR2-TLR6 heterodimers recognize triacyl and diacyl lipopeptides, respectively. In TLR2-TLR1-ligand complex, two of the three lipid chains of triacyl lipopeptide interact with TLR2, while the third chain binds to the hydrophobic channel of TLR1. Therefore, it promotes the recognition of triacyl lipopeptides. However, TLR6 lacks hydrophobic channels, so TLR2-TLR6 heterodimer does not recognize triacyl lipid peroxides. TLR5 recognizes flagellin and depends on MyD88 pathway. TLR11 identifies pathogenic bacterial components and profilin-like molecules derived from *Toxoplasma gondii*. In cytoplasm, TLR3 recognizes the dsRNA of the virus. It can bind to the N and C terminals of the convex extracellular domain of TLR3, which helps to form homologous dimers through the C terminal region, thus activating the TRIF-dependent pathway. TLR7 recognizes ssRNA and activates NF-κB and IRF7 through MyD88 to induce inflammatory cytokines and IFN I, respectively. TLR9 recognizes the CpG DNA motifs of bacteria and viruses. Downstream signal transduction requires proteolytic cleavage of TLR9 by cytoproteinase. TLR9 recruits MyD88 to activate NF-κB and IRF7. The ligand of TLR10 is unknown. The only one that uses all four adapters is TLR4, which can activate both MyD88- and TRIF-dependent pathways. TLR4 recruits TIRAP on the plasma membrane and then promotes the aggregation of MyD88, triggering NF-κB translocation to the nucleus. TLR4 then relies on endocytosis of the initiator protein and is transported to the endosomes, forming signal transduction complexes with TRAM and TRIF to initiate IRF3 activation and late TRIF-dependent pathways. The main result of TLR signal transduction is to induce the production of proinflammatory cytokines and IFN I

The two pathways have different dynamics. TLR stimulation triggers the binding of MyD88, which interacts with interleukin-1 receptor-associated kinase 4 (IRAK4) which is the most upstream serine/threonine kinase of the complex [56]. IRAK4 phosphorylates IRAK1 and separates it from the receptor complex. TRAF6 subsequently binds to phosphorylated IRAK1 [57]. The phosphorylated IRAK1 and TRAF6 separated from the receptor form complexes with transforming growth factorβ-activated kinase 1(TAK1), TAK1-binding protein 1 (TAB1) and TAB2 on the cell surface, which induce the phosphorylation of TAB2 and TAK1 [58]. IRAK1 is degraded on the cell surface, and the remaining complex including tumor necrosis factor receptor-associated factor 6 (TRAF6), TAK1, TAB1 and 2 translocates to the cytosol, resulting in ubiquitination of TRAF6 and induction of TAK1 activation. In turn, TAK1 phosphorylates mitogen-activated protein (MAP) kinase and inhibitor kappa B kinase (IKK) complexes [40]. This allows NF-κB to translocate to the nucleus and upregulate the expression of its target gene. In addition to the classical pathway dependent on MyD88, different TLRs can also transmit signals through TRIF-dependent pathway. TRIF, together with TRAF6, TRADD, Pellino-1 and RIP1, forms a multiprotein signaling complex that activates TAK1, and also activates NF-κB and mitogen-activated protein kinase (MAPK) pathways. In addition, TRIF recruits signaling complexes including non-canonical Tank-Binding-Kinase 1 (TBK1) and IKKi (IKKε) that induce the phosphorylation of interferon regulatory factor 3 (IRF3) and 7, followed by translocation to the nucleus and induction of type I IFNs [59]. The details are shown in Fig. 1.

## Role of TLRs in pathogen-induced atherosclerosis

Current epidemiological, human, and animal experiments indicate that chronic infectious diseases or biological pathogens that cause these infections are associated with progression of atherosclerotic CVD. These pathogens include *C. pneumoniae,* Periodontal pathogens*, H. pylori,* CMV, EB and the like. As major members of the host's innate immunity, TLRs play an indispensable role in the atherosclerosis caused by pathogen infection. However, there is a significant difference in the strength of the data that supports its association. Table 2 summarizes the latest research progress on how these pathogens affect the development of atherosclerosis and the types of TLRs involved.

**Table 2:** Summary of pro atherosclerotic mechanisms in chronic infections and TLRs involved

Pathogen	Phase of atherogenesis	TLRs involved
*C. pneumoniae*	Activation of endothelium	TLR2
	Migration of leucocytes	TLR4
	Lesion rupture	
	Thrombosis	
*P. gingivalis*	Activation of endothelium	TLR2
	Formation of lipid core	TLR4
	Proliferation of SMCs	
*H. pylori*	Activation of endothelium	TLR2
	Macrophage derived foam cell formation	TLR4
	Lesion instability	TLR5
		TLR9
HIV	Activation of endothelium	TLR3
	Formation of lipid core	TLR4
	Lesion rupture	TLR7
		TLR8
CMV	Activation of endothelium	TLR2
	Migration of leucocytes	TLR7
	Proliferation of SMCs	TLR9
	Lesion rupture	
	Thrombosis	
HSV	Thrombosis	TLR2
		TLR3
		TLR7
		TLR9

Summary of the aspects of pathogens affecting the development of atherosclerosis and the TLRs involved in each pathogen

*TLRs* toll like receptors, *C. pneumoniaeChlamydia pneumonia*, *P. gingivalisPorphyromonas gingivalis*, *H. pyloriHelicobacter pylori,HIV* human immunodeficiency virus, *CMV* Cytomegalovirus, *HSV* herpes simplex virus

### Chlamydia pneumoniae

*Chlamydia pneumoniae* is an obligate intracellular pathogen with unique developmental cycles and survival strategies in host cells and is an ordinary cause of community-acquired pneumonia [60, 61]. Saikku and his colleagues found for the first time that individuals with immunoglobulin G antibody specific for *C. pneumoniae* had a raised risk of MI and ASVD compared with controls [10]. Subsequently, serological correlation between antibodies to *C. pneumoniae* and atherosclerosis were reported by a series of studies [62]. The evidence of seroepidemiology has promoted the progress of animal experiments. The key issue in animal models is whether *C. pneumoniae* can induce atherosclerosis or accelerate the pathological progress of atherosclerosis. Studies have presented that *C. pneumoniae* infection promotes plaque progression in ApoE^−/−^ mice, diet-induced hyperlipidemic mice (LDLR^−/−^ and C57BL/6J mice), while *C. pneumoniae* has no effect on plaque development [63]. However, it has also been reported that researches on mice infected with *C. pneumoniae* have failed to observe the deterioration of atherosclerosis [64]. Interestingly, infection aggravated the aortic sinus lesions in C57BL/6J mice fed a high cholesterol diet, but not if infection preceded such a diet [65]. This exhibits that the atherosclerotic effect of *C. pneumoniae* infection depends on the arterial response to hyperlipidemia. A study directly inoculated *C. pneumoniae* into porcine coronary artery (damaged by balloon catheter) and pulmonary artery, resulting in thickening of the temporal lobe of the largest coronary artery, but no thickening of the pulmonary artery [66]. In addition, *C. pneumoniae* can reduce the availability of endothelial nitric oxide in ApoE^−/−^ mice, leading to endothelium-dependent relaxation damage. These experiments indicate that besides hyperlipidemia, vascular damage was identified as a prerequisite for *C. pneumoniae* infection to promote atherosclerosis. In animals with advanced atherosclerosis, *C. pneumoniae* infection did not accelerate the size of lesions, but the production of MMP-2 and MMP-9 and the decrease of fibrous cap area were observed, indicating that the degree of *C. pneumoniae* promoting lesions decreased with the progress of atherosclerosis [67]. The mechanism of *C. pneumoniae* promoting atherosclerosis is to induce chronic inflammatory response, leading to tissue injury, repair and fibrosis. Infected monocytes/macrophages can transfer *C. pneumoniae* infection to smooth muscle cells (SMCs) and ECs. *C. pneumoniae* up-regulates the expression of integrin beta 2 on the surface of macrophages, thus promoting macrophage adhesion to endothelium. ECs infected with *C. pneumoniae* can upregulate the expression of adhesion molecules such as intercellular adhesion molecule-1 (ICAM-1), VCAM-1, P-selectin and E-selectin, thereby increasing the adhesion and migration of leukocytes to endothelium [68]. In addition, Microarray analysis showed that *C. pneumoniae* infects ECs resulting in up-regulation of PDGF-B transcription, while PDGF-B contributed to SMCs proliferation and intimal thickening [69]. Coombes and his colleagues reported that repeated *C. pneumoniae* infection in normal rabbits increases intimal thickening compared with controls, and the presence of *C. pneumoniae* in aortic tissue is related with the level of PDGF-B [70]. *Chlamydia pneumoniae* promotes the activation of macrophages, ECs and SMCs through the above mechanisms.

TLRs play an important role in activating macrophages and ECs, inhibiting cholesterol outflow and promoting foaming after *C. pneumoniae* infection. The main molecules involved are TLR2 and 4. Massari et al. reported that TLR4 recognizes LPS and cHSP60 on the surface of Chlamydia [71]. Outer membrane proteins 2 (OMP2) together with cHSP60, Cpn 0980, Cpn 0809 from *C. pneumoniae* have been presented to activate macrophages via the TLR4 and MAPK pathways [72]. Paolillo et al. have proved *C. pneumoniae* can also induce the production of vascular endothelial growth factors (VEGF) and MMP-9 in human ECs via TLR2/4-dependent pathway [73]. The interaction between TLR4 and *C. pneumoniae* ligands activates the MyD88-dependent pathway in immune cells and then upregulates the expression of CD68, nitric oxide (NO), human beta-defensin-2 (HBD-2), pro-inflammatory cytokines [74]. In the presence of LDL, *C. pneumoniae* can induce foam cell formation and LDL oxidation. Cao et al. reported that foam cell formation induced by *C. pneumoniae* is modulated by TLR2 but not TLR4 [75]. However, Yoshikazu et al. demonstrated that *C. pneumoniae* can affect atherosclerotic plaque development in a TLR2/4/MyD88-dependent manner by promoting foam cell formation and enhancing activated DCs in plaques. LXR signaling can counteract *C. pneumoniae*-induced atherosclerosis by reducing cholesterol efflux and blocking TLR-dependent proinflammatory signaling [76]. In the same year, Chen et al. demonstrated *C. pneumoniae* promotes foam cell formation through activation of TLR2 and TLR4 signaling, but not TLR3, and activates downstream MyD88/TRIF-dependent pathways. They also demonstrated that LXR agonists can in turn reduce *C. pneumoniae*-mediated IRF3 activation in macrophages [77]. Futhermore, Yang et al. reported that *C. pneumoniae* can negatively regulate ABCA1 expression by the TLR2-NF-κB and miR-33 pathway in THP-1 macrophage-derived foam cells. ABCA1 is the key to macrophage cholesterol efflux and has a protective influence on atherosclerosis. Decreased expression or localization of ABCA1 on the cell membrane may lead to cholesterol accumulation in macrophages and aggravate atherosclerosis [78].

### Periodontal pathogens

Periodontal disease (PD) is a polymicrobial dysbiotic inflammatory disease with substantial inflammatory destruction of the supporting tissues, including gingival tissue, periodontal ligament, and alveolar bone. *P. gingivalis*, the best-studied bacterial pathogen associated with severe PD, is a Gram-negative anaerobic bacterium in which the host’s immune responding to the organism destroys the dental support structure [79]. A series of evidence suggests that *P. gingivalis* and periodontal disease are risk factors for diabetes, premature labor, and atherosclerotic disease and the presence of *P. gingivalis* has been detected in atherosclerotic plaques [11, 80, 81]. Velsko et al. detected *P. gingivalis* in aortic plaque by Fluorescent in-situ hybridization (FISH), and reported that chronic oral infection with *P. gingivalis* results in a specific immune response, significant increases in oral bone resorption, aortic inflammation, viable bacteria in oral epithelium and aorta, and plaque development [20]. Proinflammatory cytokine induction by *P. gingivalis* was reported to be refrained by monoclonal antibodies to TLR2 and 4, CD14 and β2 integrin [82]. Frank and his colleagues reported that *P. gingivalis* demands TLR2 to induce oral inflammatory bone loss in mice, and *P. gingivalis* infection promotes atherosclerosis in hyperlipidemia mice with increased expression of TLR2 and TLR4. Hayashi et al. proved that TLR4 has atherosclerosis protection in response to *P. gingivalis* infection [83]. In the same year, MyD88 and TRIF are proved to play important roles in the formation of foam cells induced by *P. gingivalis *[84]. *P. gingivalis* has the ability to regulate C5a signaling in neutrophils, which leads to the degradation of MyD88 by C5aR-TLR2 cross-talk involving the ubiquitin ligase Smurf1, reducing the production of inflammatory markers and resulting in reduced antimicrobial killing. Alternative TLR2 signaling through Mal and PI3 kinase (PI3K) in the absence of MyD88 to cause inflammatory activation [85]. Brown et al. demonstrated that *P. gingivalis* infection in the oral cavity can result in significant TLR2-CD36/SR-B2-mediated IL1β release and promote plaque progression [86]. HSP 60 isolated from *P. gingivalis* was confirmed to induce TLR-4 mediated ICAM-1, VCAM-1 and expression of the ox-LDL receptor-1 (LOX-1) in mice [87].

In addition to *P. gingivalis*, other significant periodontal pathogens also support the potential connection between PD and ASVD. *Treponema denticola *(*T. denticola*) is a predominantly subgingival oral spirochete closely associated with periodontal disease. Chukkapalli et al. detected *T. denticola* clusters in both gingival and aortic tissue of infected ApoE^−/−^ mice by FISH and reported that *T. denticola* infection increased alveolar bone resorption and aortic plaque area in mice, and changed the gene expression related to atherosclerosis [19]. Nussbaum et al. reported *T. denticola* cells and its major outer sheath protein (MSP) induced innate immune responses through TLR2-MyD88, whereas its lipooligosaccharide (LOS or glycolipid) induced a macrophage response through TLR4-MyD88 [88]. Recently Ruby et al. reported that *T. denticola* periplasmic flagella activate the innate immune system through TLR2 [89].

*Tannerella forsythia *(*T. forsythia*) is a Gram-negative anaerobic organism that inhabits the subgingival cavity and initiates connective tissue destruction and alveolar bone resorption in PD. Lee et al. reported *T. forsythia* and its major surface virulence factor, BspA could induce foam cell formation in THP-1 cells and accelerated the progression of atherosclerotic lesions in ApoE^−/−^ mice [90]. Ardila et al. analyzed 80 patients with chronic periodontitis and 28 healthy people and found that the levels of serum total cholesterol (TC) and LDL increases in the occurrence of *T. forsythia*, which is likely to increase the risk of atherosclerosis [91]. Chukkapalli et al. reported that *T. forsythia* infection promoted alveolar bone resorption, increased serum inflammatory markers and lipid profile changes in ApoE^−/−^ mice, but did not increase the growth of aortic plaque [92]. Onishi and his colleagues provided direct evidence for BspA binding to TLR2 and demonstrated that the release of the chemokine interleukin-8 (IL-8) from human gingival epithelial cells by BspA is TLR2-dependent [93]. These contradictory experimental results call for further research to confirm the relationship between *T. forsythia* and atherosclerosis and the role of TLRs in them.

*Fusobacterium nucleatum *(*F. nucleatum*) is another significant periodontal pathogen with oral infection. Heat-shock protein GroEL of *F. nucleatum* has been reported to induce the expression of monocyte chemokines and cell adhesion molecules, and to promote the progression of atherosclerosis in ApoE^−/−^ mice [94]. Velsko et al. discovered that *F. nucleatum* enhanced the vascular inflammation, changed the lipid profile and the gene expression of the aorta in ApoE^−/−^ mice, and significantly reduced the number of atherosclerotic plaques in the aorta, which suggested that this member of the oral microbiome has a potential protective function [95]. Recently they also discovered that in TLR2^−/−^TLR4^−/−^ mice infected with polymicrobial infection including *P. gingivalis, T. denticola, T. forsythia,* and *F. nucleatum*, atherosclerotic plaque progression was significantly reduced. However, bacterial genomic DNA was detected in multiple organs indicating an intravascular dissemination from gingival tissue to heart, aorta, kidney and lungs, suggesting that TLR2 and 4 were dispensable for systemic spread after polybacterial infections but TLR2 and 4 deficiency markedly reduces atherosclerosis induced by oral bacteria [96]. These results illustrated the role of TLR2 and 4 in atherosclerosis of periodontal bacterial infection, suggesting that focusing on periodontal disease may furnish new therapeutic options for the treatment of patients with atherosclerosis. However, a recent statement from the American Heart Association (AHA) supported an association between PD and ASVD but did not as of yet support a causal relationship. Although periodontal interventions can reduce systemic inflammation and endothelial dysfunction in short-term studies, there is still no direct evidence that they can prevent or modify ASVD progression [1].

### Gut microbiota

In addition to the oral cavity, the gut is a likely source of microorganisms that could influence ASVD. A growing body of literature showed that the permeability of oral and intestinal epithelial barrier increased, which made a small number of pathogens or its products enter the system circulation, inducing chronic low-grade inflammation and promoting inflammatory diseases including atherosclerosis [97]. Karlsson et al. found that the intestinal tract of patients with symptomatic atherosclerosis is rich in *Collinsella*, while that of healthy people is rich in *Roseburia* and *Eubacterium* by shotgun sequencing [98]. Emoto et al. identified a reduction in the operational taxonomic unit (OUT) 853 (*Bacteroides*) and an increase in the OUT 657 (*Lactobacillales*) and OUT 990 (*Clostridium subcluster XIVa*) in fecal samples of ASVD patients. The abundance of gut microorganisms such as *Bacteroides*, *Clostridium*, and *Lactobacillales* has been shown to predict ASVD, suggesting that atherosclerosis may be regulated by gut microbiota [99]. Backhed and his colleagues’ groundbreaking study on gut microbiota and lipid metabolism found that mice with normal flora could get more energy from HFD and accumulate more lipid than germ-free mice [100]. Maitra and Li discovered LPS produced by intestinal flora can significantly reduce the expression of ABCA1 and ABCG1, and inhibit the reverse transport of cholesterol [101]. Sayin et al. reported that compared with the normal mice, the gallbladder of germ-free mice was larger and the bile acid level was higher, including the difference of bile acid distribution [102]. Short chain fatty acids (SCFA) and trimethylamine oxide (TMAO) are the metabolites closely related to atherosclerosis [103]. SCFA can inhibit NF-κB and tumor necrosis factor (TNF) signaling pathway, resulting in the decrease of expression of VCAM-1 and ICAM-1, thus inhibiting the development of atherosclerosis [104]. TMAO, on the one hand, increased the expression of macrophage receptors SR-A1 and CD36, leading macrophages to phagocytize more ox-LDL, on the other hand, reduced the synthesis or transport of bile acids, reduced the reverse transport of cholesterol, thus promoting the occurrence of atherosclerosis [105, 106]. It was also reported to promote platelet activation and thus arterial thrombosis [107]. A choline analogue that has a non-lethal inhibitory effect on TMA, 3,3-dimethyl-1-butanol (DMB) was reported to be able to inhibit choline diet-enhanced endogenous macrophage foam cell formation and atherosclerotic lesion development in ApoE^−/−^ mice without alterations in circulating cholesterol levels, suggesting that targeting gut microbial production of TMA specifically and non-lethal microbial inhibitors may serve as a potential therapeutic approach for the treatment of atherosclerosis [108]. These results showed that gut microbiota affects the development of atherosclerosis mainly through three pathways: local or distant infections, regulation of cholesterol and lipid metabolism, and the effects of metabolites [109].

The commensal gut microbiota is a tonic activating factor of TLRs and other PRRs in the intestine [110]. Maitra and his colleagues confirmed high dose LPS can significantly increase the expression of NF-κB, and low dose LPS can induce macrophage inflammation and promote the occurrence of atherosclerosis through TLR4 and IRAK1 [111]. Li et al. reported that *akkermansia muciniphila* reduced the expression of CCL2 and ICAM-1 in atherosclerotic lesions, thus reducing macrophage infiltration. This effect was abolished when mice were infused with LPS with subcutaneously implanted osmotic pumps [112]. In addition to TLR4, Round et al. discovered, a symbiosis factor (PSA, polysaccharide A) of the prominent gut commensal *Bacteroides fragilis *(*B. fragilis*) signals through TLR2 directly on Foxp3(+) regulatory T cells to promote immunologic tolerance [113]. Jackel et al. reported gut microbiota regulated hepatic von Willebrand Factor (vWF) synthesis and arterial thrombus formation via TLR2 [114]. It is not clear whether ASVD can be successfully treated by targeting microbiota. Compared with the long-term use of antibiotics, it may be a new therapeutic strategy to regulate the composition of microbiota or the pathway to produce atherogenic metabolites (such as TMAO and other molecules) or to target TLR signaling pathways.

### Helicobacter pylori

*Helicobacter pylori* is a Gram-negative spiral microeosinophilic bacteria, most of which has a disposition to colonize in the gastric mucosa and cause lifelong inflammation in human gastric mucosa. It is closely related to a series of gastric diseases such as chronic gastritis, peptic ulcer, gastric mucosa-associated lymphoid tissue (MALT) lymphoma and gastric cancer [115]. In 1994, Patel and his colleagues found that the *H. pylori* infection rate in patients with coronary atherosclerosis was significantly higher than that in healthy people [116]. This report has led researchers to explore the relationship between *H. pylori* and atherosclerotic diseases. In the past 30 years, a large number of studies have confirmed its relationship with atherosclerosis, but some studies have reached contradictory conclusions. For example, Vijayvergiya et al. reported higher levels of anti-*H. pylori* IgG in the serum of CAD patients than in the control group [117]. Liu et al. conducted a meta-analysis of the relationship between *H. pylori* and MI, and found that *H. pylori* infection was significantly associated with the risk of MI [118]. However, in vivo studies conducted by Mach et al. in mice with atherosclerosis showed no evidence that *H. pylori* infection may contribute to the formation of atherosclerosis [119]. Hagiwara et al. reported that *H. pylori* DNA was not detected by detecting 50 exfoliated plaques in the neck of Japanese patients [120]. These contradictory results have led researchers to explore whether different subtypes of *H. pylori* play different roles in promoting the development of atherosclerosis. Vacuolating cytotoxin (VacA) and cytotoxin-associated gene A (CagA) protein encoded by the cytotoxin-associated genes pathogenicity island (cagPAI) are the two major *H. pylori* virulence factors [121]. Some meta-analyses suggest that CagA-positive *H. pylori* infection is more significantly associated with ASVD than CagA-negative *H. pylori* infection. Three animal studies have used *H. pylori* SS1 to inoculate gastric infections to explore whether *H. pylori* infection promotes the progression of atherosclerosis, but inconsistent results have been obtained [119, 122, 123]. Although SS1 strain is CagA-positive strain, CagY mutation lacks T4SS system, so CagA can not be effectively transferred into host. Our research team has proved that *H. pylori* PMSS1 strain, which can effectively transfect CagA into the host, was inoculated into mice with gastric infection, confirming that CagA derived from gastric epithelial cells infected by *H. pylori* promotes macrophage-derived foam cell formation and atherosclerosis [124]. Our research also confirmed that CagA protein of *H. pylori* can increase the adhesion of aortic ECs and promote atherosclerosis through NLRP3-IL1β signaling pathway. It further suggests that CagA, as a key virulence factor of *H. pylori*, is related to the occurrence and development of atherosclerosis, but the specific mechanism needs to be further studied.

Pathological studies of *H. pylori* infection mediated by TLRs mainly focus on gastric diseases. LPS derived from *H. pylori* is regarded as a direct stimulator of innate immunity mediated by TLR4. LPS of *H. pylori* activates the NF-κB pathway by ligating with TLR4, and then activates the IL-8 pathway [125]. However, some studies have shown that the production of inflammatory cytokines in gastric epithelial cells or human macrophages induced by LPS of *H. pylori* is not related to TLR4. In addition to TLR4, TLR2 has also been reported to be involved in the activation of signaling pathways after *H. pylori* infection. Triantafilou et al. demonstrated that cell activation induced by LPS from *H. pylori* and *P. gingivalis* is mediated by TLR2 and requires the formation of a heteromeric receptor complex containing TLR2, TLR1, CD36 and CD11b/CD18 [126]. Uno and his colleagues demonstrated that TLR2 of gastric epithelial cells activated Tribbles 3-NF-κB signaling pathway under the stimulation of LPS from *H. pylori*. Other studies have shown that TLR2 and 4 cooperate to cope with *H. pylori* infection. For instance, LPS of *H. pylori* can enhance the expression of TLR2 through TLR4 signal [127]. During the proliferation of gastric epithelial cells, TLR2 activated by LPS of *H. pylori* also enhanced the expression of TLR4 through MAP/ERK 1/2 kinase pathway [128]. However, other studies have shown that TLR2 and 4 work in isolation. Obonyo et al. confirmed that *H. pylori* induces IL-10 and IL-12 via TLR4/MyD88 signaling, and induces less IL-6 and IL-1β in TLR2-deficient macrophages, indicating that *H. pylori* activates TLR2 and 4, resulting in the secretion of different cytokines by macrophages [129]. In addition to TLR2 and 4, TLR5 recognizes *H. pylori*-flagellin A (FlaA). Generally speaking, TLR5 signal induces IL-8 secretion through p38-map kinase signal by recognizing bacterial flagellin. However, some studies have shown that FlaA plays a weak role in IL-8 production [130]. TLR9 has been reported to recognize unmethylated CPG DNA of *H. pylori* and produce IFN I [131]. Rad and his colleagues demonstrated that TLR9 stimulated by *H. pylori* could induce pre-inflammatory cytokines such as IL-6 and IL-12 [132]. Because of the different research background and purity of *H. pylori* and LPS, the reactivity of TLRs in gastric mucosa infected by *H. pylori* is uncertain. The role of TLRs in gastric mucosa infected by *H. pylori* provides a possible mechanism for *H. pylori* to promote the development of atherosclerosis. Whether TLRs mediate the occurrence and development of atherosclerosis in *H. pylori* infection through similar signaling pathways in gastric mucosa is uncertain, and further research is expected.

### Human immunodeficiency virus

There is increasing evidence that HIV infection and subsequent inflammation accelerate atherosclerosis. Imaging studies using coronary CTA showed that the prevalence of coronary atherosclerosis in HIV males was 59.0%, compared with 34.4% in control group [133]. Except for men with HIV, the proportion of people with non-calcified plaques was significantly higher in women with HIV than in women with control subjects (74–23%) [134]. A meta-analysis showed that there was a correlation between non-calcified coronary plaque and decreased CD4 cell count in HIV patients [135]. These clinical evidence suggests that systemic inflammation and immune activation of HIV infection contribute to the acceleration of atherosclerosis in HIV patients. Because HIV cannot replicate in rodent cells, the application of mouse models in the pathogenesis of HIV is hampered. Studies have shown that macaques infected with simian immunodeficiency virus (SIV) on an atherogenic diet show a faster deterioration of the disease, leading to an increased risk of SIV-related death [136]. However, due to the high maintenance costs, the extremely difficult genetic manipulation, and the long time of monitoring the atherosclerosis of SIV-infected monkeys, it is still difficult to apply SIV monkey model to comb the pathogenesis [137]. Animal experiments of HIV accelerating atherosclerosis still need further exploration.

TLRs seem to play an irreplaceable role in immune activation and dysfunction caused by HIV infection. It is reported that the ssRNA oligonucleotides rich in guanosine (G) and uridine (U) from HIV-1 can be specifically recognized by TLR7 and 8 [138]. Neutrophils detect HIV-1 by TLR7 and 8, which recognize viral nucleic acids. The combination of TLR7 and 8 induces the production of reactive oxygen species (ROS), which trigger network formation and lead to the elimination of network-dependent HIV-1 [139]. Stimulated by TLR7/8 agonists, the levels of cytokines/chemokines produced by DCs from HIV-infected patients were substantially higher than those from non-infected controls [140]. In addition, Meier et al. reported that plasmacytoid dendritic cells (pDCs) from women responded significantly to TLR7 ligands encoded by HIV-1 and produced more IFNα than pDCs from men, which enhanced the secondary activation of CD8 (+) T cells [141]. It suggests that regulating TLR7 pathway in pDCs might be a new method to reduce HIV-1-related pathology. Baenziger reported that HIV itself may directly lead to immune activation and dysfunction by stimulating TLR7 [142]. Borducchi et al. reported that the therapeutic vaccination with AD26/MVA (recombinant adenovirus serotype 26 (AD26) prime, modified vaccine Ankara (MVA) boost) and stimulation by TLR7 can lead to a decrease in viral DNA levels in lymph nodes and peripheral blood, and improve virological control and delay viral rebound after interruption of antiviral therapy [143]. Therefore, manipulation of TLR7 signaling may be a potential strategy to reduce chronic hyperimmune activation, thereby reducing the progression of HIV infection. Gringhuis et al. reported that the replication of HIV-1 in DCs requires TLR8 and DC-SINT signals. DC-SIGN signal is necessary to produce full-length viral transcripts [144]. In DCs, HIV-1 evades the detection of TLR8 by using the motilin protein Snapin. Inhibiting Snapin enhances the localization of early endosomes of HIV-1 and TLR8, triggers the proinflammatory response, and inhibits the infection of CD4 T cells [145]. These results show that TLR8 plays a critical role in innate immune sensing of HIV in DCs. Except for TLR7 and 8, Chen and his colleagues reported TAR RNA-containing exosomes from HIV-infected T cells activate ERK cascades in an EGFR/TLR3-dependent manner to promote the growth and progress of specific NADCs [146]. Tachado et al. demonstrated that HIV infection is associated with macrophage TLR4-mediated signal transduction impairment, especially for MyD88-dependent TLR4-mediated signal transduction pathway [147]. These findings have not yet established a link between HIV infection, TLRs and atherosclerosis, but provide a strong basis for our study on whether TLRs play a role in the process of atherosclerosis associated with HIV infection, and further research is still needed.

### Other pathogens associated with atherosclerosis

In addition to the above four pathogens, other pathogens have been identified to be associated with ASVD. For instance, CMV protein was detected in the arterial cells of atherosclerotic patients [148]. High levels of anti-CMV antibodies are associated with clinically significant atherosclerosis [149]. HCMV belongs to the beta-herpesvirus subfamily, and its chronic infection of the vasculature is associated with the development of ASVD, including atherosclerosis, restenosis, and transplant vascular sclerosis [150]. In vivo studies showed that HCMV infection of the vessel wall affects a variety of cells, including monocytes/macrophages, SMCs and ECs [151]. CMV infection mainly influences the proliferation of SMCs, leading to thickening of the intima. Another study suggests that the TLR-2 gene polymorphism may be associated with congenital CMV infection, but its mechanism remains to be elucidated [152]. Assinger et al. demonstrated HCMV binds to a TLR-2 positive platelet subpopulation, leading to signal transduction, degranulation and release of CD40L, IL-1β and VEGF [153]. pDCs is a major source of IFN-I during CMV infection. This response requires pDC-endogenous MyD88-dependent signaling by TLR7 and 9. In addition to CMV, evidence of the association between HSV and atherosclerosis was initially confirmed by Benditt et al. by in situ hybridization of atherosclerotic vessel walls [154]. In vitro studies by Hajjar et al. found that herpes infection affects the nervous system of smooth muscle cells, and excessive cholesterol ester accumulation in infected cells [155]. Sato and his colleagues found that during infection with HSV-1, TLR3 recruited the metabolic checkpoint kinase complex mtorc2 to activate the molecules needed to induce IFN I [156]. Reinert et al. reported that TLR3 plays a role in astrocytes, preventing HSV from spreading beyound the neurons that mediate the entry into the central nervous system (CNS) [157]. In addition, Ahmad et al. demonstrated that HSV-1 induced activation of TLR2-dependent IL-15 gene expression, which required recruitment of MyD88 and TIRAP/MAL and the activation of IRAK 1 and TRAF6 resulting in nuclear transfer of NF-κB [158]. Krug et al. demonstrated that the secretion of IFN I induced by HSV-1 stimulation in vitro was mediated by TLR9/MYD88 pathway [51]. Lund et al. reported that the genomic DNA of HSV-2 could bind to TLR9 and secrete interferon-alpha through pDCs [159]. Welner et al. found that the common lymphoid progenitor cells (CLPS) of mice infected with active HSV-1 tended to differentiate into DCs, which was largely dependent on TLR9 [160].

## Potential therapeutic targets

Given that innate and adaptive immunity is a major feature of atherosclerosis, it is important to consider specific strategies for immunization as a new method to the prevention and treatment of atherosclerosis. The most important challenge is to identify specific antigens associated with atherogenesis, and targeting these antigens may beneficially affect the progression of atherosclerosis. As mentioned above, many exogenous antigens have been identified in atherosclerotic plaques, including *C. pneumoniae*, *P. gingivalis*, *H. pylori*, CMV, HIV, HCV, etc. Scientists have conducted a series of antibiotic experiments against these microorganisms. In addition, vaccine experiments against these microorganisms and endogenous antigens such as LDL and apoB-100 are also underway.

### Anti-infective treatment

Muhlestein and his colleagues found that *C. pneumoniae* infection accelerated the thickness of the intimal wall and the degree of atherosclerosis in a rabbit model, and treatment with azithromycin lead to attenuation of atherosclerosis [161]. However, Rothstein et al. reported that *C. pneumoniae* infection increased the size of lesions in mice, but azithromycin did not reduce the size of aortic lesions [162]. Madan et al. have demonstrated that doxycycline treatment reduced circulating pro-inflammatory cytokine levels and the development of related atherosclerosis in ApoE^−/−^ model mice infected with *P. gingivalis *[163]. Amar et al. found that treatment with metronidazole before vaccination with *P. gingivalis* can significantly reduce the size of circulating inflammatory markers and atherosclerotic lesions [164]. Ayada et al. showed that triple drug therapy (leserazole, amoxicillin, clarithromycin) can reduce the atherogenic effect of *H. pylori* infection in gastrointestinal tract [122]. In addition to animal studies, anti-infective therapies for the treatment of atherosclerosis in humans are also underway. However, contrary to the results of animal studies, most clinical trials of anti-infective therapy did not demonstrate a role in delaying atherosclerotic events, although the antibiotic selection, treatment regimen and duration of treatment in these clinical trials are sufficient [165, 166]. The negative results of these trials indicate that although pathogens are present in atherosclerotic plaques, they may not be the indispensable culprit in the progression of atherosclerosis. However, these results do not fully confirm this point of view, because the treatment ineffectiveness of antibiotics in atherosclerotic diseases is also reasonable. First, long-term antibiotic treatment can lead to drug resistance. Secondly, it may be that antibiotic treatment is too late to produce significant clinical effects. Thirdly, persistent low-grade chronic inflammation does not respond to antibiotic treatment. Recently, a hypothesis has been proposed that infection plays a “match” role for atherosclerosis. After igniting this pathological process, the fight against this “spark” does not inhibit the progression of atherosclerosis. Therefore, the fact that anti-infective treatment is ineffective does not deny the potential link between infection and atherosclerotic disease.

### Vaccine treatment

#### Vaccine against exogenous antigens

##### Animal experiments

Given the large number of studies confirming the close association between infection and atherosclerosis, some researchers have investigated the effects of vaccination against these microorganisms on the progression of atherosclerosis. In animal models, exposure to *P. gingivalis* accelerated atherosclerosis, and its immunity reduced the extent of atherosclerosis [167]. Koizumi et al. used nasal cavity immunization with *P. gingivalis* outer membrane protein, which confirmed that the size of atherosclerotic lesions in the aortic sinus of mice was significantly reduced, and the concentration of circulating inflammatory cytokines was decreased [168]. Binder et al. confirmed that when homozygous LDLR^−/−^ mice were immunized with *S. pneumoniae*, the specific antibodies to oxidized LDL increased and atherosclerosis decreased [169]. Anti-phosphorylcholine (PC) can be produced after immunization with *S. pneumoniae*. It has been proved that immunizing ApoE^−/−^ mice with PC conjugate results in the increase of PC antibody and ox-LDL antibody and the decrease of atherosclerosis. These results suggest that immunization against exogenous antigens may be a new objective for the treatment of atherosclerosis.

##### Clinical trials

The exciting results of animal experiments have driven clinical trials to explore the role of vaccination in reducing atherosclerotic events. Lamontagne et al. conducted a hospital-based case–control study and found no hypothetical protective effect of the pneumococcal vaccination (adjusted 0.85, 95% confidence interval 0.54–1.33) in patients vaccinated 1 year before MI. Conversely, if vaccinated two or more years before admission, the correlation was stronger (adjusted to 0.33, 95% confidence interval 0.20–0.46) [170]. However, a prospective cohort study found no evidence of association between pneumococcal vaccination and reduced risk of acute myocardial infarction (AMI) (adjusted to 1.09; 95% confidence interval 0.98–1.21) or stroke (adjusted to 1.14; 95% confidence interval 1.00–1.31) [171]. Some studies have shown that influenza vaccination can reduce cardiovascular events, such as ischemia, re-admission after MI, and ASVD. A growing body of evidence supports annual influenza vaccination, leading to a reduction in ASVD, stroke and all-cause mortality in known ASVD patients; moreover, there is no proof that the vaccine has a negative impact on recipients.

#### Vaccine against endogenous antigens

In addition to vaccination against exogenous antigens, many studies have been conducted to explore vaccination against endogenous antigens. The most common research antigens are natural or modified homologous LDL and lipoproteins containing ApoB100, a major component of LDL. Because LDL has the strongest pathogenic relationship with atherosclerosis, antigens from LDL and ApoB100 are the most potent targets for vaccine development. Immunization of animals with homologous natural or ox-LDL in vaccine formulations containing different adjuvants demonstrates atherosclerotic protection [172]. However, precise mechanisms and epitopes have not been fully established until several immunoreactive peptides including p2, p143 and p210 are found to cause a 40–70% reduction in atherosclerosis and reduce plaque inflammation. P210-based vaccines have demonstrated clear atherosclerotic protection [173]. Therefore, the entire LDL particle is no longer used as an antigen, and the determination of antigenic epitopes makes more efficient vaccine development possible.

### Targeting TLRs

The role of TLRs in atherosclerosis has led researchers to explore the therapeutic potential of targeting TLRs in atherosclerosis and related diseases. Over the past decades, further attempts have been made to identify specific agonists and antagonists of TLRs. The initial clinical application of TLR therapy mainly focused on tumors and infections, but now more and more molecules are targeted at immune diseases. Animal models of atherosclerosis suggest that inhibiting TLR-dependent signals or limiting arterial exposure to PAMPS may provide therapy for the treatment or prevention of atherosclerosis [174]. Statins, as the most commonly prescribed drugs for hypercholesterolemia worldwide, may slow the progression of atherosclerosis and other inflammatory diseases by inhibiting the expression of TLR4 and regulating TLR4/MYD88/NF-κB signaling pathway [175]. Drugs that competitively inhibit the binding of intestinal bacterial LPS to TLR4/MD2 complexes, such as lipid IVa (compound 406) and Eritrean, have been reported to effectively inhibit TLR4 signaling in vitro and in vivo [176]. Since PAMPs of intestinal flora contribute to systemic inflammatory tension, treatment to modify intestinal flora is also being attempted. Prebiotic inulin has been shown to reduce LPS lumen concentration and reduce atherosclerosis in ApoE^−/−^ mice by about 35% [177]. Therefore, the possibility of regulating atherosclerosis by regulating intestinal flora should not be excluded. In a recent study, TLR4 antagonist, Rhodobacter globosa lipopolysaccharide (RS-LPS), did not alleviate the early atherosclerosis process in ApoE^−/−^ mice. However, RS-LPS reduced lesion formation in ApoE^−/−^ diabetic mice, suggesting the potential environmental dependence of TLR4 blockade on atherosclerosis [178]. A new peptide inhibitor, Viper (derived from vaccine virus protein A46), inhibits TLR4-dependent signal transduction by blocking TIR-TIR domain interactions. In addition, blocking the TLR2 signaling pathway can reduce the inflammation-promoting pathway in human atherosclerosis [179]. Opn301, an antibody against TLR2, can block TLR2-induced pro-inflammatory cytokine signaling and was reported to improve cardiac function and reduce infarct size in mice with ischemia/reperfusion (I/R) injury [180]. Most existing studies only reported the effect of TLRs on lipid accumulation, but the role of TLRs in other mechanisms of atherosclerosis, such as plaque instability and cell death, remains to be further studied. In addition, TLR plays an essential role in host defense against pathogens, and blocking TLR signals may increase the risk of infection in patients. Therefore, an appropriate risk/benefit ratio needs to be assessed. Another way to prevent TLR and its signal mediators is to focus on finding and selectively targeting atherosclerosis-related molecular patterns identified by TLRs, eliminating the risk of host defense.

## Conclusion and future perspectives

Over the past few decades, our understanding of the classical risk factors for atherosclerotic progression, the complex interactions of inflammation and immune activation has grown rapidly. There is an emerging epidemiological link between infection and ASVD. Chronic infections and atherosclerosis have a variety of biological mechanisms, and there is sufficient experimental evidence to support the atherogenic properties of various bacterial and viral infections. The most convincing evidence is that infectious agents are detected in the atherosclerotic vascular wall, and pathogen-specific antibodies are associated with atherosclerosis. These pathogens include *C. pneumoniae*, periodontal pathogens including *P. gingivalis*, *H. pylori*, CMV, EBV, HIV, HSV-1, HSV-2, and HCV. However, despite the pathophysiological correlation, clinical studies of anti-infection have not achieved satisfactory results. The reason may be due to the following three points. First, long-term antibiotic treatment can lead to drug resistance. Second, antibiotic treatment may be too late to produce significant clinical effects. Third, persistent low-grade chronic inflammation is ineffective for antibiotic treatment. Recently, a hypothesis has been proposed that infection plays a “match” role for atherosclerosis. After igniting this pathological process, the fight against this “spark” does not inhibit the progression of atherosclerosis. Therefore, the fact that anti-infective treatment is ineffective does not deny the potential link between infection and atherosclerotic disease. More research is needed to determine the exact role of microbial infections in atherosclerosis.

As the most characteristic member of PRRs, TLRs are highly evolved conserved proteins. By binding with a series of specific endogenous and exogenous ligands, it plays an important role in innate immune mechanism. TLR signaling pathway can be broadly classified as a MyD88-dependent pathway that drives the production of inflammatory cytokines and a TRIF-dependent pathway responsible for the production of IFN I and inflammatory cytokines. The role of TLRs in the development of related atherosclerosis has just begun to be discovered. Both critical and resident vascular cells express multiple TLRs in the initiation and progression of atherosclerosis, suggesting that these receptors and their ligands are key players in atherosclerosis. Many experiments have proved that TLRs are involved in signal pathways that affect the progression of atherosclerosis after microbial infection. For example, TLR2 and 4 play an important role in activating macrophages and ECs after *C. pneumoniae* infection, inhibiting cholesterol outflow and promoting foam formation. *P. gingivalis* infection can accelerate atherosclerosis in hyperlipidemic mice and increase the expression of TLR2 and 4. LPS derived from *H. pylori* is regarded as a direct stimulator of innate immunity mediated by TLR4. The crucial role of TLRs in gastric mucosa infected by *H. pylori* provides a possible mechanism for *H. pylori* to promote the development of atherosclerosis. TLRs seem to also play an irreplaceable role in immune activation and dysfunction caused by HIV infection. Overall, the activation of TLR 2 and 4 seems to provide a new mechanism for infection-related atherosclerosis.

In summary, this review highlights the role of TLRs in the pathogenesis of atherosclerosis associated with microbial infections, particularly *C. pneumoniae*, periodontal pathogens, *H. pylori* and CMV. In our efforts to reduce atherosclerosis, determining the right target pathway has been our Achilles heel. Is it the microbe itself or the myriad of signaling pathways, including TLRs that are activated in chronic infections, which ultimately lead to atherosclerosis? More research is needed to demonstrate the hypothesis that pathogens contribute to atherosclerosis through TLR activation. Identification of pathogenesis of microbial induction may reveal drug targets for therapeutic intervention or prevention of atherosclerosis through vaccines or immunomodulatory therapy. Since the direct role of these pathogens in atherosclerosis remains uncertain, it is unclear whether vaccines against these microorganisms are useful strategies for treating human atherosclerosis. In this conjecture, the development of atherosclerotic vaccines for various signaling pathways associated with infection may be promising.
